# Predictive Value of Classical and Emerging Autoantibodies for Cardiac Dysfunction in Systemic Sclerosis: Systematic Review

**DOI:** 10.3390/jcm14186383

**Published:** 2025-09-10

**Authors:** Mislav Radić, Tina Bečić, Petra Šimac, Hana Đogaš, Ivana Jukić, Damir Fabijanić, Josipa Radić

**Affiliations:** 1 Department of Internal Medicine, Division of Rheumatology, Allergology and Clinical Immunology, University Hospital of Split, 21000 Split, Croatia; petra_simac@hotmail.com; 2 Internal Medicine Department, School of Medicine, University of Split, 21000 Split, Croatia; josiparadic1973@gmail.com; 3 Department of Cardiovascular Diseases, University Hospital of Split, 21000 Split, Croatia; tina.becic@gmail.com (T.B.); damirfabijanic62@gmail.com (D.F.); 4 Department of Neurology, University Hospital of Split, 21000 Split, Croatia; hana.dogas@gmail.com; 5 Department of Internal Medicine, Division of Gastroenterology, University Hospital of Split, 21000 Split, Croatia; ivjukic@gmail.com; 6 Faculty of Health Sciences, University of Split, 21000 Split, Croatia; 7 Clinical Propedeutics Department, School of Medicine, University of Split, 21000 Split, Croatia; 8 Department of Internal Medicine, Division of Nephrology, Dialysis and Arterial Hypertension, University Hospital of Split, 21000 Split, Croatia

**Keywords:** myocardial fibrosis, arrhythmias, anti-topoisomerase I, anti-centromere antibody, cardiac biomarkers, cardiac imaging

## Abstract

**Background:** Cardiac involvement is a major cause of morbidity and mortality in systemic sclerosis (SSc). Autoantibodies may help identify patients at increased cardiovascular (CV) risk. This systematic review aimed to assess the predictive value of classical and emerging SSc-related autoantibodies for cardiac involvement and their integration with imaging and cardiac biomarkers. **Methods:** A comprehensive literature search was conducted in PubMed, Web of Science, Scopus, and the Cochrane Library up to 16 July 2025. Studies were included if they reported associations between specific autoantibodies and cardiac outcomes (e.g., myocardial fibrosis, conduction abnormalities, arrhythmias, ventricular dysfunction) in adult patients with SSc. Data extraction and quality assessment followed PRISMA 2020 guidelines. The review protocol was registered in PROSPERO (registration ID: CRD420251107782). **Results:** Anti-topoisomerase I antibodies were associated with myocardial fibrosis, subclinical systolic and diastolic dysfunction, elevated cardiac biomarkers, and pathological findings on cardiac magnetic resonance imaging. Anti-centromere antibodies were linked to conduction system abnormalities, particularly among older individuals. Anti-RNA polymerase III and anti-U3 ribonucleoprotein antibodies correlated strongly with arrhythmias and pericardial involvement. Novel autoantibodies, such as anti-heart antibodies and anti-intercalated disk antibodies, were linked to early myocardial injury, although their clinical utility requires further validation. Across studies, serological markers alone were insufficient to predict cardiac outcomes without concurrent imaging or biomarker evaluation. **Conclusions:** Autoantibody profiling plays an important role in CV risk stratification in SSc. Combining serological testing with cardiac biomarkers and advanced imaging enhances early detection and supports individualized monitoring. Further longitudinal studies are needed to validate predictive models and optimize patient outcomes.

## 1. Introduction

Systemic sclerosis (SSc) is a rare autoimmune disease whose main feature is progressive fibrosis of the skin and internal organs [1]. Myocardial fibrosis is a life-threatening condition, as confirmed by postmortem autopsy findings showing fibrotic myocardial abnormalities in up to 80% of patients with SSc [2]. In addition to fibrosis, other cardiac manifestations, including arrhythmias, conduction abnormalities, valvular involvement, and pericardial disease, represent clinically significant complications of SSc. The clearest available data are about arrhythmias, which are the cause of death in 6% of patients [3]. Real-life world data have established an incidence of 255 per 10,000 person-years (2.55% annually) for any new arrhythmias in SSc. In the same study, it was reported that SSc patients had significantly higher rates of conduction disorders compared to controls, with an incidence rate ratio (IRR) of approximately 2.1 [4]. Although valvular and pericardial abnormalities are recognized as important cardiac manifestations in SSc, data regarding their incidence and prevalence remain limited. Among valvular pathologies, mitral regurgitation appears to be the most common, being reported in 5.2% of cases, followed by aortic stenosis (3.5%) and aortic regurgitation (1.7%) [5]. Pericardial involvement, primarily in the form of pericardial effusion, has been reported in 15% to 43% of patients with SSc [6]. A high prevalence of subclinical arrhythmias and conduction system disturbances, particularly among patients with diffuse cutaneous (dc) SSc, was also reported. Notable findings included sinus tachycardia, premature ventricular contractions (PVCs), and atrioventricular (AV) blocks, some of which were asymptomatic [7]. Undoubtedly, these disorders contribute to increased morbidity and mortality in affected individuals. Timely detection of cardiac involvement, especially the recognition of patients at higher risk of developing these manifestations remains a clinical challenge. Research efforts are aimed at discovering and specifying biomarkers that may improve early detection and current therapy options [1].

The generally accepted classification of cardiac involvement in SSc distinguishes between SSc-primary cardiac involvement (SSc-pHI) and secondary cardiac involvement. SSc-pHI is thought to arise directly as a consequence of the pathophysiological disorders of the disease, while secondary complications are associated with other systemic manifestations of SSc. These manifestations include the very common pulmonary arterial hypertension (PAH), followed by interstitial lung disease (ILD) and the slightly rarer renal scleroderma crisis [8,9]. SSc-pHI includes the aforementioned manifestations such as myocardial dysfunction, conduction system disturbances, various arrhythmias, as well as pericardial and valvular involvement [1]. Electrocardiographic (ECG) and cohort-based studies reveal considerable variability in the reported prevalence of arrhythmias in SSc. Atrial fibrillation is observed in approximately 5–9% of patients, while ventricular arrhythmias may occur in up to 16%. Premature ventricular contractions are detected in 22–55% of patients on Holter monitoring, and non-sustained ventricular tachycardia is reported in 8–30%, depending on the diagnostic method employed [10,11].

It is hypothesized that alterations of the blood vessel walls lead to SSc-pHI complications. More precisely, inflammation of the endothelium, accompanied by fibrosis and vasculopathy is considered to be substantial underlying pathophysiological mechanism. All of these changes occur in both small and large blood vessels simultaneously [12,13]. According to data from the European League Against Rheumatism Scleroderma Trials and Research (EUSTAR) registry, up to 12% of deaths in SSc are related to myocardial dysfunction, conduction system aberrations, arrhythmias, and impairment of the pericardium and valves [14]. Secondary cardiovascular (CV) involvement, including ischemic cardiomyopathy, also represents a significant clinical burden in SSc, given that these complications are a major cause of morbidity and mortality. Approximately 26% of deaths related to SSc are attributed to cardiac complications [6]. Differentiating between certain disorders can at times be challenging, highlighting the ongoing need to revise and refine diagnostic guidelines and recommendations. SSc-pHI is relatively common and may occur in both the limited cutaneous (lc) and dcSSc subsets [15]. Given the heterogeneity of cardiac involvement, clinical symptoms are highly variable. The vast majority of patients initially present with nonspecific symptoms such as mild dyspnea, fatigue, palpitations, and fluctuating blood pressure. This is why it is sometimes difficult to recognize SSc-pHI in the early stages [16].

Emerging evidence indicates that SSc-specific autoantibodies may play a significant role in development of cardiac damage. This systematic review aims to explore and compare classical and emerging autoantibodies associated with cardiac complications in SSc, highlighting the clinical relevance of cardiac involvement and serological profiles in the disease.

## 2. Methods

### 2.1. Protocol, Registration and Criteria

This systematic review was conducted in accordance with the Preferred Reporting Items for Systematic Reviews and Meta-Analyses (PRISMA 2020) guidelines [17]. The review protocol was registered in the International Prospective Register of Systematic Reviews (PROSPERO; registration ID: CRD420251107782).

We included peer-reviewed studies that involved the following:

- Enrolled adult patients diagnosed with SSc based on American College of Rheumatology/European League Against Rheumatism (ACR/EULAR) criteria [18];
- Reported cardiac involvement (e.g., myocardial fibrosis, arrhythmias, conduction abnormalities, left ventricular (LV) dysfunction);
- Assessed classical or emerging autoantibodies (e.g., anti-centromere antibodies [ACA], anti-topoisomerase I antibodies [anti-Scl-70], anti-RNA polymerase III [anti-RNAP III], anti-U3 ribonucleoprotein [anti-U3 RNP], anti-heart antibodies [AHA], anti-intercalated disk antibodies [AIDA]);
- Used imaging (e.g., cardiac magnetic resonance [CMR], echocardiography) or biomarker-based methods (e.g., N-terminal pro-brain natriuretic peptide [NT-proBNP], troponin) for cardiac assessment.

Exclusion criteria:

- Non-human studies, editorials, and conference abstracts.

### 2.2. Information Sources

The following electronic databases were systematically searched:

- PubMed
- Web of Science
- Scopus
- Cochrane Library

The final search was performed on 16 July 2025.

### 2.3. Search Strategy and Study Selection

The search strategy included both controlled vocabulary and free-text terms, such as (“systemic sclerosis” OR “scleroderma”) AND (various autoantibodies, including anti-topoisomerase I [anti-Scl-70], anti-centromere [ACA], anti-RNA polymerase III [anti-RNAP III], and anti-U3 RNP antibodies) AND (cardiac involvement terms such as “myocardial fibrosis,” “arrhythmias,” “conduction abnormalities,” “left ventricular [LV] dysfunction,” “NT-proBNP,” and “troponin”). No language or date restrictions were applied initially. Reference lists of the included studies and relevant reviews were manually screened to identify additional articles. Two independent reviewers (TB and PS) screened titles and abstracts, followed by a full-text review. Discrepancies were resolved through discussion or adjudicated by a third reviewer (MR). The selection process was documented using a PRISMA 2020 flow diagram.

### 2.4. Data Collection Process

A standardized data extraction form was used. The following data were collected:

- Study design and setting;
- Sample size and demographics;
- Diagnostic criteria for SSc;
- Autoantibody types and titers;
- Cardiac outcome measures (e.g., fibrosis, LV dysfunction, arrhythmias);
- Imaging and biomarkers used;
- Main findings and statistical associations.

### 2.5. Risk of Bias Assessment

Risk of bias was assessed using the Newcastle-Ottawa Scale (NOS) for observational cohort studies [19]. Each study was rated independently by two reviewers. Studies scoring ≥ 7 were considered to have low risk of bias while those scoring from 4 to 6 were considered to have moderate risk of bias.

### 2.6. Synthesis of Results and Assessment of Evidence Quality

Due to variability across studies in design, patient populations, definitions of cardiac involvement, and methods used to assess autoantibodies, a narrative synthesis approach was used. Findings were reviewed and summarized descriptively, with results organized by autoantibody type, type of cardiac manifestation (such as myocardial fibrosis, conduction disturbances, or arrhythmias), and the diagnostic tools applied (including imaging techniques and cardiac biomarkers). This method allowed for a structured interpretation of the evidence and identification of clinically relevant patterns across diverse study settings. As this review was based on a qualitative synthesis, the quality and consistency of the included evidence were evaluated narratively. Key factors considered included the methodological design of each study, risk of bias, clarity and consistency in the definition of cardiac outcomes, and the reliability of autoantibody assessment. Particular attention was given to the strength and consistency of reported associations, the reproducibility of findings across different cohorts, and any notable methodological limitations that could affect interpretation.

## 3. Results

### 3.1. Study Selection

**Figure 1:**
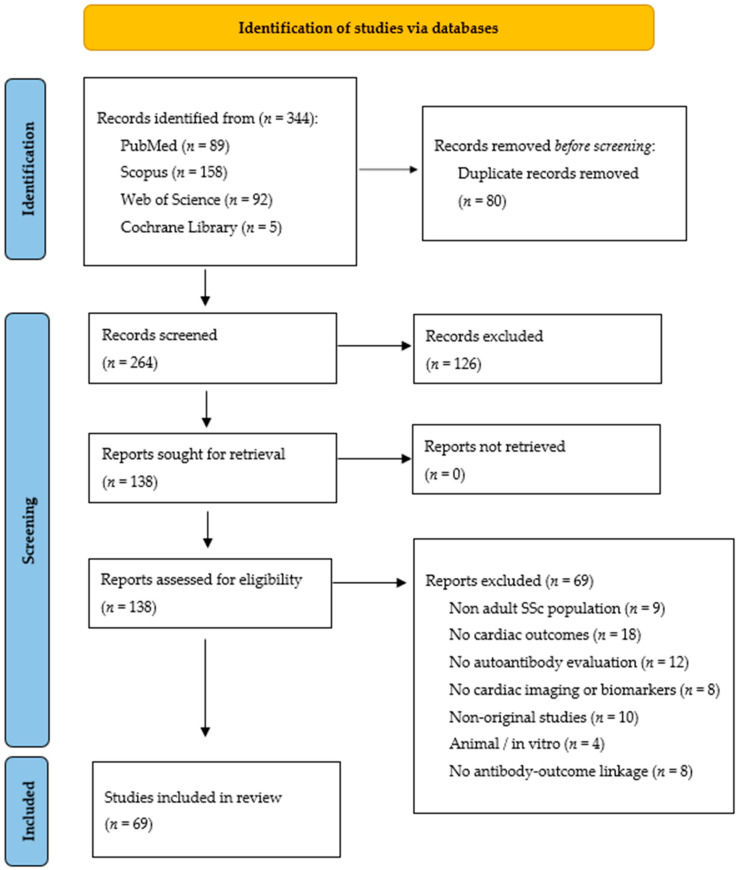
Flowchart illustrating the study selection process, developed in accordance with the PRISMA 2020 (Preferred Reporting Items for Systematic Reviews and Meta-Analyses) guidelines.

The initial database search identified a total of 344 records: 89 from PubMed, 92 from Web of Science, 158 from Scopus, and 5 from the Cochrane Library. After removing duplicates, 264 records remained for title and abstract screening. Of these, 126 were excluded based on irrelevance to the predefined inclusion criteria. A total of 138 full-text articles were assessed for eligibility. Of these, 69 were excluded for the following reasons: non-adult SSc populations (*n* = 9), absence of cardiac outcomes (*n* = 18), lack of autoantibody evaluation (*n* = 12), no cardiac imaging or biomarker data (*n* = 8), non-original studies such as conference abstracts or commentaries (*n* = 10), studies involving animal or in vitro models (*n* = 4), and incomplete linkage between autoantibody data and cardiac outcomes (*n* = 8). Ultimately, 69 studies were included in the qualitative synthesis. A meta-analysis was not performed due to substantial clinical and methodological heterogeneity, including differences in study design, patient populations, cardiac outcome definitions, and diagnostic methods used to assess autoantibodies. The PRISMA 2020 flow diagram illustrating the selection process is presented in Figure 1.

### 3.2. Study Characteristics and Quality Assessment

Of the 69 studies included, 20 were observational studies reporting on the association between specific autoantibodies and cardiac involvement in SSc which were eligible for formal quality assessment using the NOS. The NOS evaluates the quality of cohort and case-control studies across three domains: selection, comparability, and outcome/exposure. A score of 7 or higher was considered indicative of high methodological quality [18]. Among these, 16 studies (80%) were rated as high quality (NOS score ≥ 7), while 4 (20%) were considered to be of moderate-to-low quality (NOS score < 7). The majority of studies were cohort designs (*n* = 15), including registry-based, population-based, single-center, and multicenter cohorts. Additionally, three studies used cross-sectional or cardiac imaging-based observational methodologies, and one employed a case-control design. High-quality studies frequently involved large patient populations, use of national or multicenter registries, defined cardiac endpoints, and appropriate adjustments for confounding variables. Notable examples include registry-based studies by Bairkdar et al. (2024) and Allanore et al. (2010), or cross-sectional observational study by Höppner et al. (2023)**,** each achieving the highest NOS score of 9 [4,20,21]. Conversely, lower-scoring studies often lacked comparison groups, had smaller sample sizes, shorter follow-up, or incomplete reporting of outcome definitions and potential confounders. Overall, the quality of evidence was judged to be methodologically robust, lending credibility to the narrative synthesis and supporting the observed associations between autoantibody profiles and CV manifestations in SSc. Table 1 summarizes the methodological quality assessment of the included studies.

## 4. Pathogenesis of Primary Cardiac Involvement in SSc

The pathogenesis of SSc-pHI is multifactorial and includes activation of the immune system, dysfunction of the microvasculature, and ultimately progressive fibrosis. It is believed that myocardial damage in SSc is a consequence of chronic ischemia secondary to microvascular injury and sustained inflammation. These processes lead to myocardial cell damage and fibrotic remodeling. Even in the absence of manifest cardiac symptoms, histopathological hallmark features such as patchy myocardial fibrosis, perivascular inflammatory infiltrates, and small-vessel obliteration can be found in patients with SSc [11,34]. Vascular dysfunction is the initial event in SSc. It occurs as a result of early endothelial cells damage and disruption of vasodilation and angiogenesis, accompanied by persistently elevated levels of oxidative stress [35]. Ultimately, these changes cause chronic hypoxia and ischemia reperfusion injury, which leads to cardiomyocyte death and replacement fibrosis [36]. Pivotal profibrotic mediators in these signaling pathways include transforming growth factor-beta (TGF-β), endothelin-1, connective tissue growth factor (CTGF), and platelet-derived growth factor (PDGF). Their predominant role is to stimulate fibroblast activation, myofibroblast transdifferentiation, and extracellular matrix deposition [37,38]. There is growing evidence of immune-mediated mechanisms that significantly contribute to cardiac involvement. Dysregulation of both innate and adaptive immunity sustains chronic tissue inflammation and fibroblast activation. This particularly involves dysfunction of Th17 cells and hyperactivation of B cells and plasmacytoid dendritic cells [39]. Pro-inflammatory cytokines, interleukin (IL)-6 and -1, are linked with myocardial fibrosis and conduction disorders. Their pathogenic effect is thought to be mediated through the activation of local fibroblasts and inflammasome signaling pathways [40,41]. Recent studies have highlighted endothelial-to-mesenchymal transition (EndMT) as a novel pathogenic mechanism in SSc. It involves the transformation of endothelial cells into mesenchymal-like cells, contributing directly to the activation of fibroblasts within cardiac and vascular tissues [42,43]. Epigenetic alterations, including micro-ribonucleic acid (microRNA) dysregulation and aberrant histone modifications, may facilitate profibrotic gene expression profiles in the myocardium. A meta-analysis of 16 studies demonstrated that differences in microRNA expression may influence the vasculopathy and fibrosis in SSc, and yet can serve as potential biomarkers [39,44]. In conclusion, the complex pathophysiology of SSc-pHI is primarily driven by vascular injury, immune system activation, and fibrotic processes.

## 5. Autoantibodies and Cardiac Manifestations in SSc

Determining the serological profile is essential in the diagnosis, classification, and prognosis of SSc. Traditionally, ACA and anti-Scl-70 are used to define clinical subsets and predict the risk of organ involvement [1,45]. Other autoantibodies associated with cardiac manifestations in SSc include the following: anti-RNAP III anti-U3 RNP or anti-fibrillarin, anti-Ku antibodies, anti-histone antibodies, anti-Th/To antibodies, AHA, AIDA, anti-U1 ribonucleoprotein antibodies (anti-U1 RNP), anti-Pm/Scl antibodies (anti-PmScl), anti-Ro/Sjögren’s-syndrome-related antigen A antibodies (anti-Ro/SSA), anti-PL-7 and anti-PL-12 antibodies (anti-PL-7/anti-PL-12), anti–nucleolar organizing region 90 antibodies (anti-NOR90), and anti-Mi-2 antibodies [41]. Although the strength of evidence varies for each individual autoantibody, their roles in cardiac manifestations of SSc will be discussed below, with primary focus on the two most clinically relevant: ACA and anti-Scl-70.

### 5.1. Anticentromere Antibodies

ACA are classically associated with lcSSc and a generally more indolent disease course [45]. ACA, found in about 30% of SSc patients, are directed against centromeric proteins that play a role in chromosome segregation [45,46]. For ACA positivity, the most characteristic feature is the presence of Raynaud’s phenomenon (RP) and a higher risk of developing PAH that is not related to ILD [45,47]. SSc patients with decline in diffusing capacity of the lungs for carbon monoxide (DLCO) and higher pulmonary artery pressure values have lower overall survival rate [45,48]. In general, ACA positivity is rarely linked to serious cardiac complications. According to real-world data, this does not appear to be applicable to conduction system disorders. Results from a Brazilian observational study demonstrated that ACA positivity was significantly associated with cardiac conduction blocks: AV block and bundle branch block [49]. Despite limited sample size of the aforementioned study, results indicate a possible pathogenic role of ACA in disrupting cardiac conduction system, potentially through mechanisms such as microvascular ischemia or autoimmune-mediated myocardial damage. Conduction abnormalities may arise even without overt cardiac symptoms, underscoring the importance of regular ECG and Holter monitoring [32]. Importance of regular cardiac monitoring is evident in the results of a SSc patient’s cohort study followed over 26 years, which showed a statistically significant difference in the occurrence of conduction disorders between SSc and non-SSc subjects. Moreover, conduction abnormalities, including first-degree AV block and bundle branch block, were present in approximately 15% of SSc patients, regardless of clinical presentation. In contrast, only 7% of non-SSc subjects exhibited such cardiac abnormalities. Therefore, it can be concluded that SSc represents a significant risk factor for development of conduction system disorders, regardless of disease duration [22]. Furthermore, ACA positivity is associated with a higher prevalence of conduction disturbances and arrhythmias in patients with lcSSc. Subclinical sinoatrial and AV conduction delays have been documented in approximately 5% to 7% of asymptomatic ACA-positive individuals, particularly in older adults [50]. These findings support the rationale for routine ECG surveillance in this population [51]. In addition to arrhythmias, ACA have also been associated with LV dysfunction, although they do not represent an independent predictor of left ventricular diastolic dysfunction (LVDD) [23]. Based on findings from a cardiopulmonary exercise testing (CPET) cohort, ACA were more prevalent among SSc patients with respiratory limitation and least common in those with LV dsyfunction [52]. Together, these data highlight the need for early and proactive CV evaluation in ACA-positive individuals, even in the absence of cardiopulmonary symptoms.

### 5.2. Anti-Topoisomerase I Antibodies

Anti-Scl-70 antibodies are directed against deoxyribonucleic acid (DNA) topoisomerase I, an enzyme essential for DNA replication and repair. These antibodies are highly specific for SSc [53,54]. Anti-Scl-70 are most commonly associated with dcSSc and have been strongly linked to increased fibrosis, including myocardial fibrosis [11,55,56]. This fibrotic burden correlates with elevated serum levels of NT-proBNP and a heightened risk of structural cardiac abnormalities, including arrhythmias and conduction disturbances [20,56]. Patients with this antibody profile frequently demonstrate early cardiac involvement, including both systolic and diastolic dysfunction and myocardial perfusion defects [26,52,57]. In the EUSTAR registry, LV dysfunction was identified in 5.4% of SSc patients, with anti-Scl-70 antibodies showing a modest but statistically weak association. Stronger independent predictors included older age, male sex, digital ulcers, myositis, and pulmonary involvement [20]. Several studies have demonstrated that anti-Scl-70 positivity correlates with a subclinical myocardial involvement. Specifically, myocardial fibrosis can be detected by CMR with late gadolinium enhancement (LGE) and T1 mapping techniques. This fibrosis is often accompanied by an increased risk of ventricular arrhythmias and conduction disturbances even in asymptomatic patients [27,56]. Patchy myocardial fibrosis has been documented in approximately 56% to 66% of SSc patients with anti-Scl-70 antibodies. This fibrotic burden occurs significantly more often than in ACA-positive cases and frequently coexists with microvascular perfusion defects despite preserved LV ejection fraction [11,28,30]. Although the study included 75% of patients who were positive for anti-Scl-70 antibodies, and 65% of them had elevated troponin I levels, no direct statistical analysis was performed to assess the association between anti-Scl-70 positivity and CV outcomes [29]. Rising anti–Scl-70 titers have also been shown to parallel worsening cardiac biomarkers and imaging abnormalities, supporting their use as prognostic tools for longitudinal monitoring [24]. Long-term follow-up studies confirm that anti-Scl-70 positivity is associated with a higher incidence of major CV events, including heart failure and arrhythmias. In a prospective cohort with three years of follow-up, Dumitru et al. reported that anti-Scl-70-positive patients had an approximately threefold increased risk of developing LGE-detected myocardial fibrosis, arrhythmias, and elevated NT-proBNP levels compared to those without the antibody. Beyond structural imaging, anti-Topo I titers have also shown associations with disease activity, severity, and cardiac biomarker elevation [25]. In addition to the aforementioned association with LV dysfunction, there is evidence that anti–Scl-70 antibodies are associated with subclinical right ventricular myocardial dysfunction and PAH [33].

### 5.3. Emerging Autoantibodies and Cardiac Manifestations in SSc

In recent years, attention has turned toward emerging autoantibodies for their potential to enhance CV risk stratification in SSc. Although the results of numerous studies did not find a significant association between specific autoantibodies and cardiac involvement, some cohorts have nevertheless noted the influence of SSc-associated antibodies and cardiac involvement [58]. Anti-RNAP III antibodies, targeting subunits of the ribonucleic acid (RNA) polymerase III complex, are recognized for their association with rapidly progressive skin thickening and scleroderma renal crisis. Several case-series and registry reports have also linked anti-RNAP III positivity to pericardial effusion, cardiac tamponade, and arrhythmias, as well as fibrotic and inflammatory myocardial changes [31,59]. Cohort studies on anti-U3 RNP antibodies have demonstrated strong associations with myocardial fibrosis and conduction system disease, particularly among African-American patients. It was found that anti-fibrillarin positivity, present in 18.5% of African-American SSc patients, was significantly associated with higher rates of pericarditis and broader internal organ involvement, suggesting a link to cardiac manifestation [60]. Antifibrillarin antibodies target fibrillarin, a nucleolar protein essential for ribosomal RNA processing. These antibodies are most frequently observed in patients with dcSSc and have been strongly associated with more severe cardiac involvement, including arrhythmias and conduction abnormalities [21]. Steen et al. first described the link between antifibrillarin antibodies positivity and diffuse disease phenotype [61].

While each of these antibodies provides valuable insights into disease phenotype, few studies have comprehensively compared their utility in predicting cardiac dysfunction [62]. For instance, the German cohort of SSc patients with positive anti-Scl-70 antibodies had a slightly higher prevalence of pathological ECG findings, as well as conduction disturbances, which was not found in the Danish cohort of patients [63,64]. Furthermore, in a large sample of subjects, it was determined that anti-Scl-70 and anti-U3 RNP antibodies positivity is associated with a higher prevalence of more severe heart disorders compared to positive ACA, anti-RNAP III, anti-U1 RNP, anti-PmScl or anti-Th/To antibodies [65]. These results were not confirmed in the UK cohort of patients. Moreover, in these patients there was no association of cardiac manifestations and SSc antibodies [55]. Some authors have associated anti-Ku, anti-histone, and anti-RNA polymerase (I, II, and III) antibodies with a higher risk of heart disease and noted that anti-Th/To antibodies may be more strongly associated with pericarditis than ACA [55,63,66]. All of these data indicate the extreme heterogeneity of the disease itself, making it particularly difficult to outline specific conclusions that could apply to the vast majority of patients. Table 2 summarizes analyzed autoantibodies and their impact on the CV system, as well as their clinical relevance for patient monitoring. Table 3 summarizes SSc-related autoantibodies and the qualitative strength of evidence supporting their association with cardiac involvement using explicit categorical labels instead of colors. The grading (high, moderate, preliminary, low to minimal) reflects consistency and reproducibility of reported associations across observational studies and registries rather than quantitative effect size.

### 5.4. Associations Between Autoantibody Profiles and Cardiac Outcomes

Several cohort studies have explored the association between SSc-specific autoantibodies and cardiac involvement, although direct comparisons remain limited. Data from the large French national cohort (*n* = 3528) revealed that approximately 11% of patients developed new-onset cardiac manifestations, including LV systolic dysfunction and LVDD, within five years, with higher risk observed among patients with dcSSc, a phenotype commonly associated with anti-Scl-70 [67]. Similarly, analyses from the EUSTAR database, comprising more than 17,000 patients, identified predictors of primary myocardial involvement, though specific autoantibody stratification was not consistently reported [68].

In contrast, studies evaluating classic autoantibody profiles (such as ACA and anti-Scl-70) have produced inconsistent associations with cardiac pathology, including myocardial fibrosis, systolic or diastolic dysfunction, and arrhythmias [10]. These findings imply that traditional serological antibodies alone may be insufficient predictors of CV outcomes in SSc [60,69]. Additional insights from the Oslo SSc cohort and other longitudinal studies emphasize the relevance of integrating autoantibody testing with cardiac biomarkers and imaging. Elevated NT-proBNP and troponin have been repeatedly observed in dcSSc, frequently associated with anti-Scl-70, correlating with myocardial fibrosis, conduction abnormalities, and subclinical LV dysfunction [70].

Most recent data from the EUSTAR database demonstrated that anti-Scl-70 antibodies are independently associated with the onset of primary heart involvement in patients with SSc [10]. In contrast, ACA positivity, though less frequently implicated in primary myocardial pathology, has been correlated with increased risk of PAH, particularly in older SSc patients [71]. AHA and AIDA antibodies intend to have a potential pathogenic role in myocardial fibrosis and other cardiac disorders. These antibodies can be found in up to 49% and 56% of patients, respectively. Their presence is often associated with elevated troponin levels, arrhythmias, and reduced LV function. These antibodies tend to appear more frequently in patients who exhibit conduction abnormalities, diastolic dysfunction, or myocardial fibrosis on CMR, suggesting a possible role in early myocardial injury [72,73].

Notably, cardiac manifestations in SSc often develop within the first three to five years of disease onset and can remain subclinical for a long period. Factors such as older age at diagnosis, male sex, and comorbidities like hypertension or diabetes seem to increase both the likelihood and severity of cardiac complications [20]. While current evidence remains limited, early treatment with immunosuppressive agents or vasodilators may help reduce inflammation and slow the progression of fibrosis, particularly in patients identified early through autoantibody testing and cardiac biomarkers [10]. Altogether, combining serologic, imaging, and clinical information could significantly improve early detection and CV risk stratification in SSc.

Additionally, when it comes to the association of SSc-related antibodies with LV systolic dysfunction and LVDD, a recently published meta-analysis has demonstrated their involvement in the development of these complications. Specifically, anti-Scl-70 positivity is associated with both diastolic and systolic dysfunction, while anti-RNA polymerase III and anti-U3 RNP positivity are associated with systolic dysfunction [74].

Recent evidence suggests that ACA are strong predictors of conduction system disease, while anti-Topo I antibodies are associated with myocardial fibrosis and dysfunction. Anti-RNAP III and ANA-negative subtypes have also been linked to arrhythmic and fibrotic manifestations. Newly identified myocardial-specific antibodies offer even greater cardiac specificity [11,22,31,32,50,55,56,59,72,73]. Integrating laboratory findings with clinical symptoms will unquestionably facilitate earlier diagnosis of cardiac involvement, thereby improving the quality of life for SSc patients and ultimately reducing mortality.

Although formal guidelines on screening frequency for cardiac involvement in asymptomatic patients are lacking, a recent expert consensus from the World Scleroderma Foundation (WSF) and the Heart Failure Association (HFA) offers practical recommendations to guide clinical decision-making. The consensus suggests that all patients should undergo an initial CV assessment that includes detailed medical history, physical examination, resting ECG, transthoracic echocardiography, and measurement of cardiac biomarkers such as troponin and NT-proBNP. If abnormalities are detected or clinical suspicion is high, additional testing such as CMR, Holter monitoring, or stress imaging may be appropriate, with decisions tailored to the individual through multidisciplinary discussion. Although the panel did not recommend fixed screening intervals, regular follow-up, typically once a year, was encouraged, especially for patients at higher risk or those with abnormal baseline findings [75].

Diagnosing and managing patients with SSc requires close collaboration between rheumatologists and cardiologists, as multidisciplinary approach plays a key role in reducing both morbidity and mortality. Looking ahead, emerging serum biomarkers may provide improved specificity and hold promise as more reliable indicators of CV risk in this population.

Tools like SCORE (Systematic Coronary Risk Evaluation), FRS (Framingham Risk Score), and RSS (Reynolds Risk Score) are widely used to estimate CV risk in the general population and can offer some guidance when evaluating patients with SSc. However, their applicability in SSc is limited. These tools were not designed to account for the unique features of autoimmune diseases, such as chronic systemic inflammation, immune-mediated vascular damage, and microvascular dysfunction. All of these factors contribute to CV complications in SSc. As a result, they may significantly underestimate actual risk in this patient group. While efforts are ongoing to develop adapted risk models that include inflammatory and immunological markers, such tools are still in development and have yet to be validated for routine use in SSc care [76]. The importance of timely detection of cardiac abnormalities in SSc is further supported by findings from a recently published pilot study, in which the authors highlighted the potential of CMR imaging to identify patients who may benefit from closer monitoring and individualized therapeutic strategies [77].

Despite inconsistent and certainly insufficient data, it can be presumed that autoantibody profiling in SSc may provide critical insight into the risk of cardiac involvement. Furthermore, these findings underscore the importance of integrating autoantibody status with clinical, imaging, and biomarker data to strengthen cardiac risk stratification in SSc. Additionally, routine antibody screening may help guide referral for cardiac imaging or rhythm monitoring, particularly in patients positive for anti-Scl-70, anti-RNA polymerase III, anti-U3 RNP, or anti-Ro52, which appear to be the most promising candidates for incorporation into future risk stratification algorithms [19,23,28,35]. However, it is important to emphasize that the current evidence remains insufficient to mandate antibody-driven screening strategies in clinical practice.

It is important to acknowledge key limitations of the current evidence. First, the available studies are highly heterogeneous in design, ranging from small cross-sectional analyses to retrospective cohorts, often with limited follow-up duration. Second, cardiac outcomes are inconsistently defined and measured, with differences in imaging modalities, biomarkers, and diagnostic criteria, making cross-study comparisons challenging. Third, patient populations vary widely in sample size, ethnic background, and clinical subsets (limited vs. diffuse cutaneous disease), further contributing to heterogeneity. Finally, standardized protocols for cardiac assessment in SSc are lacking, and many studies are underpowered to detect clinically meaningful associations.

## 6. Future Research Directions

Future large, prospective, multicenter studies are needed to strengthen confirmation of the associations between SSc-related autoantibodies and cardiac outcomes by applying standardized and widely available methods. Combining antibody profiles with cardiac imaging techniques such as echocardiography and CMR, together with circulating biomarkers including NT-proBNP and troponin, may support the development of multiparametric models for risk prediction and stratification [12,15,18,33]. Among the various autoantibodies, anti-Scl-70, anti-RNA polymerase III, anti-U3 RNP, and anti-Ro52 appear to be the most promising candidates for incorporation into future risk algorithms, as they consistently associate with myocardial fibrosis, arrhythmias, and pulmonary hypertension [19,23,28,35]. Moreover, longitudinal studies are required to determine whether early identification of high-risk antibody profiles can guide personalized surveillance and enable earlier therapeutic interventions to improve patient outcomes.

## 7. Conclusions

Autoantibody profiling in SSc provides meaningful insights into the risk of cardiac involvement, particularly in identifying patients at higher risk for myocardial fibrosis, conduction abnormalities, and arrhythmias. While certain autoantibodies, such as anti-Scl-70, ACA and anti-U3 RNP, demonstrate clear associations with specific cardiac phenotypes, their diagnostic and prognostic value is greatest when interpreted in conjunction with cardiac imaging and biomarker data. Future research should prioritize well-designed, multicenter longitudinal studies that integrate serological profiles with clinical and imaging findings to refine CV risk assessment and improve patient outcomes.

## Figures and Tables

**Table 1:** NOS (Newcastle-Ottawa Scale) assessment of the included studies.

Study (First Author, Year)	Reference No.	Study Design	NOS Score	Risk of Bias
Bairkdar et al., 2024	[4]	Cohort	9	Low
Muresan et al., 2016	[7]	Cohort	7	Low
Tzelepis et al., 2007	[11]	Cohort	8	Low
Allanore et al., 2010	[20]	Cohort	9	Low
Höppner et al., 2023	[21]	Cross-sectional Observational	9	Low
Radwan et al., 2021	[22]	Cohort	8	Low
Lopez Nunez et al., 2023	[23]	Cross-sectional	8	Low
Avouac et al., 2015	[24]	Cohort	8	Low
Dumitru et al., 2021	[25]	Cohort	8	Low
Cusma Piccione et al., 2013	[26]	Cohort	7	Low
Barison et al., 2015	[27]	Cross-sectional observational	7	Low
Hachulla et al., 2009	[28]	Cohort	7	Low
Krumm et al., 2016	[29]	Cross-sectional cohort	7	Low
Kobayashi et al., 2009	[30]	Cohort	7	Low
Hui et al., 2021	[31]	Cohort	7	Low
Vacca et al., 2014	[32]	Cohort	7	Low
D’Andrea et al., 2004	[33]	Cohort	6	Moderate

**Table 2:** Overview of systemic sclerosis-related autoantibodies, associated cardiac manifestations, and their clinical relevance.

Autoantibody	Associated Cardiac Manifestations	Clinical Utility
Anti-centromere (ACA)	Conduction abnormalities, arrhythmias (esp. in older adults); potential risk of PAH	Common marker for lcSSc; useful for PAH risk but limited in predicting fibrosis
Anti-topoisomerase I (anti-Scl-70)	Myocardial fibrosis, diastolic dysfunction, elevated NT-proBNP and troponin, arrhythmias	High-risk marker for early cardiac fibrosis and dysfunction; strong clinical relevance
Anti-RNA polymerase III	Pericardial effusion, arrhythmias, potential tamponade	Predictive for scleroderma renal crisis and cardiac effusion
Anti-U3 RNP (anti-fibrillarin)	Myocardial fibrosis, conduction system disease, pericarditis (especially in African-American patients)	Ethnic-specific marker (African-American); strong indicator of cardiac involvement
Anti-Ku/Anti-Histone	Increased risk of heart disease	Supportive marker; less specific
Anti-Th/To	Greater association with pericarditis compared to ACA	More specific than ACA for pericardial involvement
AHA/AIDA	Potential myocardial specificity; associated with cardiac fibrosis	Emerging biomarkers; require further validation
Anti-U1 RNP	Mild conduction system disease; may coexist with PAH and overlap syndromes	Overlap syndromes (e.g., MCTD); cardiac risk less well-defined
Anti-PmScl	Occasional myocardial involvement; more often linked with myositis-overlap	Useful in overlap syndrome diagnostics; less specific for cardiac disease
Anti-Ro/SSA	Occasionally pericardial effusion; mostly linked with systemic autoimmune overlap	Non-specific marker; may aid in overlap diagnosis
Anti-PL7/PL12	Rarely cardiac involvement; more associated with inflammatory myopathy	More relevant in ILD and myositis; cardiac links unclear
Anti-NOR90	Limited evidence for direct cardiac involvement	Rare; not clinically routine for cardiac screening
Anti-Mi-2	Primarily associated with dermatomyositis; occasional myocardial fibrosis	Muscle-specific; used in differential diagnosis with myositis-cardiac overlap

Abbreviations: ACA: anti-centromere antibodies; AHA: anti-heart antibodies; AIDA: anti-intercalated disk antibodies; anti-Scl-70: anti-topoisomerase I antibodies; anti-U3 RNP: anti-U3 ribonucleoprotein (anti-fibrillarin); anti-U1 RNP: anti-U1 ribonucleoprotein; anti-Th/To: anti-Th/To ribonucleoprotein antibodies; anti-PmScl: anti-polymyositis/scleroderma overlap antibodies; anti-PL7/PL12: aminoacyl-tRNA synthetase antibodies; anti-Mi-2: antibodies targeting Mi-2 helicase; anti-NOR90: anti-nucleolar organizer region 90 antibodies; dcSSc: diffuse cutaneous systemic sclerosis; ILD: interstitial lung disease; lcSSc: limited cutaneous systemic sclerosis; MCTD: mixed connective tissue disease; PAH: pulmonary arterial hypertension.

**Table 3:** Summary of systemic sclerosis–related autoantibodies and the qualitative strength of evidence supporting their association with cardiac involvement.

Autoantibody	Strength of Evidence for Cardiac Involvement
Anti–Scl-70	High
ACA	Moderate
Anti–RNAP III	Moderate
Anti–U3 RNP	Moderate
AHA/AIDA	Preliminary
Others (Ku, histone, Th/To, U1 RNP, PmScl, Ro/SSA, PL7/PL12, NOR90, Mi-2)	Low to minimal

Abbreviations: ACA: anti-centromere antibodies; AHA: anti-heart antibodies; AIDA: anti-intercalated disk antibodies; anti-Scl-70: anti-topoisomerase I antibodies; anti-RNAP III: anti-RNA polymerase III; anti-U3 RNP: anti-U3 ribonucleoprotein (anti-fibrillarin); anti-PmScl: anti-polymyositis/scleroderma overlap antibodies; anti-Ro/SSA: anti-Ro/Sjögren’s-syndrome-related antigen A antibodies; anti-NOR90; anti-nucleolar organizer region 90 antibodies; anti-Mi-2: antibodies targeting Mi-2 helicase; PL7: anti-PL7 antibodies; PL12: anti-PL-12 antibodies.

## Data Availability

Data is available on request to corresponding author.

## Abbreviations

ACA	anti-centromere antibodies
ACR/EULAR	American College of Rheumatology/European League Against Rheumatism
AHA	anti-heart antibodies
AIDA	anti–intercalated disk antibodies
Anti-NOR90	anti–nucleolar organizing region 90 antibodies
anti-Mi2	antibodies targeting Mi-2 helicase
anti-PL-7	anti–PL7 antibodies
anti-PL12	anti–PL-12 antibodies
anti-PmScl	anti–Pm/Scl antibodies
anti-RNAP III	anti–RNA polymerase III
anti-Ro/SSA	anti–Ro/Sjögren’s-syndrome-related antigen A antibodies
anti-Scl-70	anti–topoisomerase I antibodies
anti-U1 RNP	anti–U1 ribonucleoprotein antibodies
anti-U3 RNP	anti–U3 ribonucleoprotein
AV	atrioventricular
CMR	cardiac magnetic resonance
CPET	cardiopulmonary exercise testing
CTGF	connective tissue growth factor
CV	cardiovascular
dc	diffuse cutaneous
DLCO	diffusing capacity of the lungs for carbon monoxide
DNA	deoxyribonucleic acid
ECG	Electrocardiographic
EndMT	endothelial-to-mesenchymal transition
EUSTAR	European League Against Rheumatism Scleroderma Trials and Research
FRS	Framingham Risk Score
HFA	Heart Failure Association
IL	interleukin
ILD	interstitial lung disease
IRR	incidence rate ratio
lc	limited cutaneous
LGE	late gadolinium enhancement
LV	left ventricular
LVDD	left ventricular diastolic dysfunction
microRNA	micro-ribonucleic acid
NOS	Newcastle–Ottawa Scale
NT-proBNP	N-terminal pro–brain natriuretic peptide
PAH	pulmonary arterial hypertension
PDGF	platelet-derived growth factor
PRISMA	Preferred Reporting Items for Systematic Reviews and Meta-Analyses
PVCs	premature ventricular contractions
RNA	ribonucleic acid
RP	Raynaud’s phenomenon
RSS	Reynolds Risk Score
SCORE	Systematic Coronary Risk Evaluation
SSc	systemic sclerosis
SSc-pHI	SSc-primary cardiac involvement
TGF-β	transforming growth factor-beta
WSF	World Scleroderma Foundation
